# PCIF1 drives oesophageal squamous cell carcinoma progression via m6Am‐mediated suppression of MTF2 translation

**DOI:** 10.1002/ctm2.70286

**Published:** 2025-03-28

**Authors:** Kang Li, Yuxuan Yi, Rongsong Ling, Caihua Zhang, Zhihui Zhang, Yue Wang, Ganping Wang, Jie Chen, Maosheng Cheng, Shuang Chen

**Affiliations:** ^1^ Otorhinolaryngology Hospital, Center for Translational Medicine The First Affiliated Hospital Sun Yat‐sen University Guangzhou China; ^2^ Department of Radiation Oncology Sun Yat‐sen University Cancer Center, State Key Laboratory of Oncology in South China, Collaborative Innovation Center of Cancer Medicine Guangzhou China; ^3^ Hospital of Stomatology, Guangdong Provincial Key Laboratory of Stomatology, Guanghua School of Stomatology Sun Yat‐Sen University Guangzhou China; ^4^ Department of Urology, Zhujiang Hospital Southern Medical University Guangzhou China

**Keywords:** m6Am modification, MTF2, OSCC, PCIF1

## Abstract

Oesophageal squamous cell carcinoma (OSCC) represents a highly aggressive malignancy with limited therapeutic options and poor prognosis. This study uncovers PCIF1 as a critical driver of OSCC progression via m6Am RNA modification, leading to translational repression of the tumour suppressor MTF2. Our results demonstrate that PCIF1 selectively suppresses MTF2 translation, activating oncogenic pathways that promote OSCC growth. In vitro and in vivo models confirm that PCIF1 knockdown reduces OSCC progression, whereas MTF2 knockdown counteracts this effect, highlighting the importance of the PCIF1‐MTF2 axis. Clinical analyses further reveal that high PCIF1 expression and low MTF2 expression correlate with advanced tumour stage, poor treatment response and decreased overall survival. Furthermore, in a preclinical mouse model, PCIF1 knockout enhanced the efficacy of anti‐PD1 immunotherapy, reducing tumour burden and improving histological outcomes. Notably, flow cytometry analysis indicated that PCIF1 primarily exerts its effects through tumour‐intrinsic mechanisms rather than direct modulation of the immune microenvironment, distinguishing its mode of action from PD1 blockade. These findings establish PCIF1 and MTF2 as promising prognostic markers and therapeutic targets for OSCC, offering new avenues for treatment strategies and patient stratification.

**Key points:**

- PCIF1 promotes OSCC progression via m6Am methylation at the MTF2 mRNA 5′ cap.
- m6Am methylation suppresses MTF2 translation, enhancing tumour cell proliferation and invasion.
- Targeting PCIF1 holds therapeutic potential for OSCC treatment.

## Graphical abstract

1. PCIF1 promotes OSCC progression via m6Am methylation at the MTF2 mRNA 5′ cap.
2. m6Am methylation suppresses MTF2 translation, enhancing tumour cell proliferation and invasion.
3. Targeting PCIF1 holds therapeutic potential for OSCC treatment.

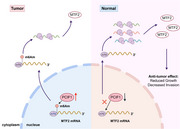

## 1 INTRODUCTION

Globally, oesophageal squamous cell carcinoma (OSCC) ranks among the most aggressive malignancies,^1^ particularly in high‐incidence regions such as Eastern Asia.^2^, ^3^ Despite advancements in multimodal treatment strategies, including surgery, chemotherapy, radiotherapy and emerging immunotherapies,^4^, ^5^, ^6^ with a 5‐year overall survival rate of less than 20%, the prognosis for OSCC remains dismal.^7^, ^8^ This dismal outcome is primarily due to late‐stage diagnosis, high metastatic potential, frequent recurrence and intrinsic resistance to conventional therapies. These challenges underscore the urgent need to elucidate the molecular mechanisms driving OSCC progression, with the ultimate goal of identifying novel therapeutic targets that could improve clinical outcomes.

Post‐transcriptional modifications have been increasingly recognized in recent studies as pivotal in cancer biology, with RNA methylation playing a central role in regulating gene expression.^9^, ^10^ Among RNA modifications, N6,2′‐O‐dimethyladenosine (m6Am) has gained particular prominence. Located immediately at the first transcribed nucleotide near the 7‐methylguanosine (m7G) cap, m6Am influences mRNA stability and translation,^11^, ^12^, ^13^ thereby regulating vital cellular processes including proliferation, differentiation and survival. Phosphorylated CTD‐interacting factor 1 (PCIF1) has been identified as the sole methyltransferase catalyzing m6Am modification.^14^, ^15^ By modulating m6Am levels, PCIF1 critically impacts mRNA stability and translation, contributing to oncogenesis.^12^, ^16^, ^17^ Despite increasing evidence supporting the oncogenic role of m6Am modification, its precise impact on OSCC progression remains largely unexplored. Given that RNA modifications can influence drug resistance, metastasis and tumour microenvironment (TME) interactions,^18^ understanding how PCIF1‐mediated m6Am modifications contribute to OSCC malignancy may provide novel therapeutic targets for intervention.

PCIF1‐mediated m6Am modification has been shown to promote tumourigenesis in several cancers, including colorectal cancer, glioblastoma and gastric cancer, by enhancing the stability and translation of oncogenic mRNAs.^12^, ^13^, ^16^, ^19^ In these cancers, PCIF1 overexpression correlates with poor prognosis and increased tumour aggressiveness. However, the functional role of PCIF1 in OSCC remains poorly characterized, and its specific downstream targets in this context have not been elucidated. Furthermore, the interplay between m6Am modifications and resistance to immunotherapy has not been well studied, despite growing evidence that epitranscriptomic alterations can impact immune evasion. Given the emerging role of m6Am in tumour biology, investigating the contribution of PCIF1 to OSCC progression is critical to advancing our understanding of the molecular drivers of this disease.

As a critical component of the polycomb repressive complex 2 (PRC2), metal response element binding transcription factor 2 (MTF2) is vital for chromatin remodelling and preserving genomic stability.^20^, ^21^, ^22^ Functioning as a tumour suppressor, MTF2 mediates gene silencing through PRC2‐driven H3K27me3 trimethylation, effectively repressing oncogene transcription.^23^ Dysregulation of MTF2 has been implicated in tumourigenesis in diverse cancers, including leukaemia and colorectal cancer,^24^, ^25^ where its loss leads to aberrant gene expression and chromatin instability. However, the functional role of MTF2 in OSCC, as well as the mechanisms underlying its potential suppression, remains insufficiently explored.

In this study, we hypothesize that PCIF1 drives OSCC progression by suppressing MTF2 translation through m6Am modification. By downregulating MTF2, PCIF1 is proposed to facilitate oncogenic behaviours, including enhanced cell proliferation, migration and invasion. To test this hypothesis, in vitro and in vivo assays were performed to evaluate the regulatory effect of PCIF1 on MTF2 levels and its functional consequences in OSCC progression. Moreover, we investigated whether PCIF1 knockout could potentiate the therapeutic efficacy of anti‐PD1 immunotherapy by alleviating tumour aggressiveness, offering a novel approach to enhancing immunotherapy response. By elucidating the PCIF1‐MTF2 axis, this study uncovers a novel oncogenic mechanism in OSCC and establishes the foundation for targeting PCIF1 as a promising therapeutic approach in this aggressive malignancy.

## 2 MATERIALS AND METHODS

### 2.1 Patient sample

The study incorporated samples collected from OSCC patients undergoing surgical resection at Sun Yat‐sen University Cancer Center (Table S1) and The First Affiliated Hospital of Sun Yat‐sen University (Table S2). Written informed consent was obtained from all patients before sample collection. Tumour tissues and adjacent normal tissues were obtained immediately after resection and then fixed in neutral‐buffered formalin for subsequent processing. The research was carried out with the approval of the Ethics Committee at Sun Yat‐sen University.

### 2.2 Cell culture

KYSE30, KYSE150 and the immortalized oesophageal epithelial cell line SHEE were obtained from the American Type Culture Collection. TE1, TE10 and TE12 were procured from Wuhan Pricella Biotechnology. Cells were maintained in Dulbecco's modified Eagle medium (TransGen Biotech, AQ601) containing 10% foetal bovine serum (FBS, A3160801; GIBCO), 1% penicillin‐streptomycin (15140122; GIBCO) at 37°C in a humidified atmosphere with 5% CO_2_. Regular mycoplasma detection was conducted throughout the study to ensure the quality of the cell lines.

### 2.3 Cell transfection

For gene knockdown experiments, short guide RNAs (sgRNAs) targeting PCIF1 were designed and cloned into a lentiviral vector system. The specific sequences of the sgRNAs used for PCIF1 knockdown are available in Table S3. Cells were transfected with sgRNA oligonucleotides targeting MTF2 or a control sgRNA to achieve MTF2 knockdown. The sequences are detailed in Table S3.

The catalytically inactive PCIF1 mutant (amino acid residues 160–163, LFPD mutated to AFPA) was provided by Tianhua Zhou from Zhejiang University School of Medicine, Department of Cell Biology and Gastroenterology. The PCIF1 overexpression plasmid was generated by cloning the full‐length open reading frame of the human PCIF1 gene (NM_022104.3) into the pLVX expression vector (GeneCopoeia). Wild‐type or mutant PCIF1 plasmids were introduced into cells using Lipofectamine 2000 (11668019; Invitrogen).

### 2.4 Transgenic mouse model construction

To generate a transgenic mouse model with specific knockout of PCIF1 in oesophageal epithelial cells, we used several mouse strains. *K14^CreER^* mice were sourced from The Jackson Laboratory, whereas *Pcif1^flox^* mice were obtained from Biocytogen Pharmaceuticals and *Mtf2^flox^* mice from Shanghai Model Organisms Center, Inc. For conditional knockout induction, adult mice were administered tamoxifen (T5648; Sigma) intraperitoneally at a dose of 75 mg/kg/day for five consecutive days. Successful gene deletion was confirmed by PCR analysis of oesophageal tissues. To induce OSCC, 6‐week‐old mice received drinking water containing 50 µg/mL 4‐nitroquinoline 1‐oxide (4NQO, N8141; Sigma) for a period of 16 weeks. The 4NQO stock solution was prepared at 5 mg/mL in propylene glycol and diluted before administration. For immunotherapy experiments, an anti‐PD1 monoclonal antibody (BE0146; BioXcell) was administered intraperitoneally at a dose of 200 µg per mouse every 2 days, starting at Week 22 and continuing until Week 26. Control group mice were administered an equivalent volume of IgG isotype control antibody. All animal experiments in this study were approved by the Institutional Animal Care and Use Committee of Sun Yat‐sen University.

### 2.5 Nude mice xenograft tumourigenesis

Male BALB/c nude mice (6 weeks old) were injected subcutaneously with 1 × 10^6^ KYSE150 OSCC cells stably expressing either PCIF1 sgRNA or control sgRNA. Tumour dimensions were recorded every 3 days using calipers, and volumes were calculated with the formula (length × width^2^)/2. Mice were sacrificed on Day 30, and the tumours were collected for subsequent examination.

### 2.6 Western blotting

OSCC cell proteins were extracted using RIPA buffer (P0013B; Beyotime) with added protease inhibitors. The bicinchoninic acid (BCA, 23227; ThermoFisher) assay was used to measure protein concentrations. Proteins were separated on sodium dodecyl sulfate‐polyacrylamide gel electrophoresis (SDS‐PAGE) gels in equal amounts and transferred to polyvinylidene fluoride (PVDF) membranes. These membranes were incubated with primary antibodies against PCIF1 (ASE‐39532; SAB), MTF2 (16208‐1; Proteintech), GOLIM4 (31083‐1; Proteintech) and GAPDH (10494‐1; Proteintech), followed by HRP‐conjugated secondary antibodies (SA00001; Proteintech). Protein detection was carried out using an enhanced chemiluminescence system.

### 2.7 Quantitative real‐time PCR (qPCR)

Total RNA extraction from OSCC cells was performed using TRIzol reagent (15596018; ThermoFisher) per the manufacturer's protocol. RNA concentration and purity were measured using a NanoDrop spectrophotometer. For reverse transcription, 1 µg of RNA was converted to cDNA using a reverse transcription kit (R323‐01; Vazyme). qPCR analysis, using SYBR Green Master Mix (Q711‐02; Vazyme) on a real‐time PCR system, quantified PCIF1 and MTF2 expression, with GAPDH as the internal control. Primer details are provided in Table S3.

### 2.8 Polysome profiling

KYSE150 cell lines with or without PCIF1 knockout were utilized for polysome profiling. Cells were pre‐treated with cycloheximide (HY‐12320; MCE; 100 µg/mL) to stabilize ribosome–mRNA complexes, followed by lysis in polysome lysis buffer. A 10%–50% sucrose gradient was used to separate the cell lysates by ultracentrifugation at 36 000 rpm for 2 h. RNA was extracted from the collected polysome‐associated fractions. qPCR was used to assess MTF2 mRNA levels, comparing the PCIF1 control group with the PCIF1 knockout group.

### 2.9 MTF2 5′UTR luciferase reporter assay

To explore the role of PCIF1 in regulating MTF2 translation, the 5′UTR WT (where WT is wild type) and 5′UTR mut (mutant) of MTF2 was cloned into a luciferase reporter construct. The 5′UTR WT construct contains the full wild‐type sequence of MTF2, whereas the 5′UTR mut construct includes mutations at the key regulatory sites. To assess luciferase activity, OSCC cells were co‐transfected with 5′UTR WT or 5′UTR mut reporter constructs along with a control plasmid, a PCIF1 overexpression plasmid or a catalytically inactive PCIF1 mutant plasmid. Forty‐eight hours post‐transfection, luciferase activity was assessed using a dual‐luciferase reporter assay, with Renilla luciferase serving as the normalization control.

### 2.10 Actinomycin D assay

To evaluate the stability of MTF2 mRNA, OSCC cells were exposed to actinomycin D (A432787; Aladdin) to inhibit new RNA synthesis. Cells were harvested at multiple time points, typically at 0‐, 6‐ and 12‐h post‐treatment, to monitor the decay of MTF2 mRNA. MTF2 mRNA levels were assessed at different time points through qPCR.

### 2.11 m6Am sequencing

Total RNA isolation from OSCC cells was performed with TRIzol reagent as per the manufacturer's guidelines. The extracted RNA (∼10 µg) was fragmented to obtain ∼100–200 nt fragments using RNA fragmentation buffer (E6150S; NEB) at 70°C for 7.5 min. The fragmented RNAs were then subjected to m7G immunoprecipitation using anti‐m7G antibodies (RN017 M; Medical & Biological Laboratories) conjugated to pierce protein A/G magnetic beads at 4°C for 3 h. The m7G‐enriched RNA was eluted, followed by a second round of immunoprecipitation with anti‐m6A antibodies (202003; Synaptic Systems) under similar conditions to enrich for m6Am‐containing fragments. After stringent washing, the antibody‐bound RNA was extracted using TRIzol reagent and ethanol‐precipitated overnight. Library preparation was carried out with the NEBNext Ultra II Directional RNA Library Prep Kit using the enriched RNA, and sequencing was conducted on the Illumina HiSeq4000 platform.

### 2.12 m6Am peak calling

The sequenced reads obtained from m6Am‐Seq were first trimmed for quality and adapters using Trim Galore. Cleaned reads were aligned to the hg38 version of the human genome with STAR. Aligned BAM files were used to identify m6Am peaks using exomePeak2. A false discovery rate threshold (FDR < .05) was used to filter peak calling results and ensure statistical significance.

### 2.13 Motif discovery

To identify potential motifs associated with m6Am peaks, the sequences surrounding the called peaks were extracted using BEDTools. Motif discovery was performed using HOMER, which was set to identify enriched motifs within a ±50 nt window around the m6Am sites.

### 2.14 Colony formation assay

In six‐well plates, 500 OSCC cells per well were cultured under standard conditions for 14 days to facilitate colony formation. The colonies were then fixed with methanol, stained with crystal violet (.1%) and counted manually.

### 2.15 Transwell migration and invasion assay

Transwell chambers with an 8.0 µm pore size (Corning) were used to assess cell migration and invasion. For migration, 2 × 10^4^ cells in serum‐free medium were added to the upper chamber, with the lower chamber containing medium with 20% FBS. For invasion, matrigel‐coated inserts were used to mimic the extracellular matrix. After 24 h, migrated or invaded cells on the lower surface of the membrane were fixed, stained with crystal violet and microscopically counted.

### 2.16 Cell apoptosis detection

To assess apoptosis, OSCC cells were harvested, rinsed in Phosphate buffer saline (PBS) and subjected to staining with an Annexin V‐FITC/PI Apoptosis Detection Kit (70‐AP107; MultiSciences). Following a 15‐min incubation in the dark at room temperature, the cells underwent analysis on a flow cytometer. Annexin V‐positive cells indicate apoptosis, whereas propidium iodide staining indicates cell membrane permeability.

### 2.17 Immunohistochemistry (IHC) staining

Sections from patient‐derived samples and xenograft tumours were prepared for IHC staining. Deparaffinized and rehydrated sections were treated with sodium citrate buffer (pH 6.0) at high pressure for 15 min to retrieve antigens. To inhibit endogenous peroxidase activity, 3% hydrogen peroxide was applied for 10 min at room temperature. Then sections were blocked with 5% bovine serum albumin for 30 min. Primary antibodies specific to PCIF1 (NBP2‐13740; Novus), Ki67 (NB500‐170; Novus) and MTF2 (16208‐1; Proteintech) were applied and incubated overnight at 4°C. Slides were washed and treated with biotinylated secondary antibodies at room temperature for 30 min. Visualization was performed with a streptavidin–HRP complex and DAB chromogen, followed by haematoxylin counterstaining, dehydration, clearing and mounting. Microscopic evaluation was performed to quantify staining intensity and localization.

### 2.18 Haematoxylin and eosin (H&E) staining

Tissue sections from formalin‐fixed, paraffin‐embedded patient specimens and xenograft tumours were utilized for H&E staining. Deparaffinization with xylene, rehydration through graded ethanol and rinsing in distilled water were performed before staining with haematoxylin for 5 min. Sections were washed, differentiated with acid alcohol, blued with alkaline water and counterstained for 2 min with eosin. After dehydration in ethanol, clearing with xylene and mounting, sections were observed with a light microscope for histological analysis.

### 2.19 RNA immunoprecipitation (RIP)

For assessing the interaction between PCIF1 and MTF2 mRNA, OSCC cells were lysed in RIP lysis buffer containing protease and RNase inhibitors. The lysate was incubated overnight at 4°C with PCIF1 antibody (98085S; Cell Signaling Technology) conjugated to Protein A/G magnetic beads. RNA from the immunoprecipitated complexes was extracted using TRIzol reagent following washing. qPCR was performed to measure MTF2, GAPDH and GOLIM4 mRNA levels. IgG served as a negative control, and input RNA was used for normalization.

### 2.20 m6A‐RIP qPCR

RNA was isolated using TRIzol Reagent (15596018; ThermoFisher), followed by mRNA purification with the magnetic mRNA isolation kit (S1550S; NEB). For m6A immunoprecipitation, 2 µg of m6A antibody (202003; Synaptic Systems) was coupled to protein A/G magnetic beads (88803; ThermoFisher) in IP buffer (150 mM NaCl, 10 mM Tris‐HCl, pH 7.4,. 1% NP‐40) and incubated at 4°C for 2 h. Ten percent of the mRNA was reserved as input, and the remaining portion (∼2 µg) was incubated with the antibody‐bead complex in 500 µL IP buffer containing 200 U RNase inhibitor (EO0381; ThermoFisher) at 4°C for 2 h. The enriched m6A RNA was eluted twice with 100 µL elution buffer (150 mM NaCl, 10 mM Tris‐HCl, pH 7.4,. 1% NP‐40, 200 U RNase inhibitor) and recovered by ethanol precipitation. Both input and m6A‐enriched RNA were subjected to qRT‐PCR analysis.

### 2.21 Flow cytometry analysis

Tumour tissues were collected from mice and processed into single‐cell suspensions by enzymatic digestion with collagenase IV (07909; Stemcell) and DNase I (E127‐01B; NovoProtein), followed by filtration through a 70‐µm cell strainer. The resulting single‐cell suspensions were stained with anti‐mouse CD3 APC (20–0032; Tonbo; 1:200), anti‐mouse CD8a FITC (35–0081;Tonbo; 1:200), anti‐Fpr1 (NLS1878; Novus) and anti‐GZMB PE (12‐8898‐82;Invitrogen; 1:200). Stained cells were analyzed using a flow cytometer, and data were processed with CytExpert software. CD8^+^ T cells were gated from CD3^+^ populations, and the expression of FPR1 and GZMB within CD8^+^ T cells was quantified.

### 2.22 Statistical analysis

Unless stated otherwise, experiments were carried out in triplicate, and data were presented as mean ± standard deviation (SD). GraphPad Prism 9 was used for statistical analyses. The unpaired, two‐tailed Student's *t*‐test was used to compare two groups, whereas one‐way analysis of variance followed by Tukey's multiple comparison test was applied for comparisons across multiple groups. Survival analyses were conducted using Kaplan–Meier curves and differences in survival rates were determined by log‐rank tests.

## 3 RESULTS

### 3.1 Comprehensive analysis of PCIF1 expression and its prognostic significance in OSCC

**FIGURE 1:**
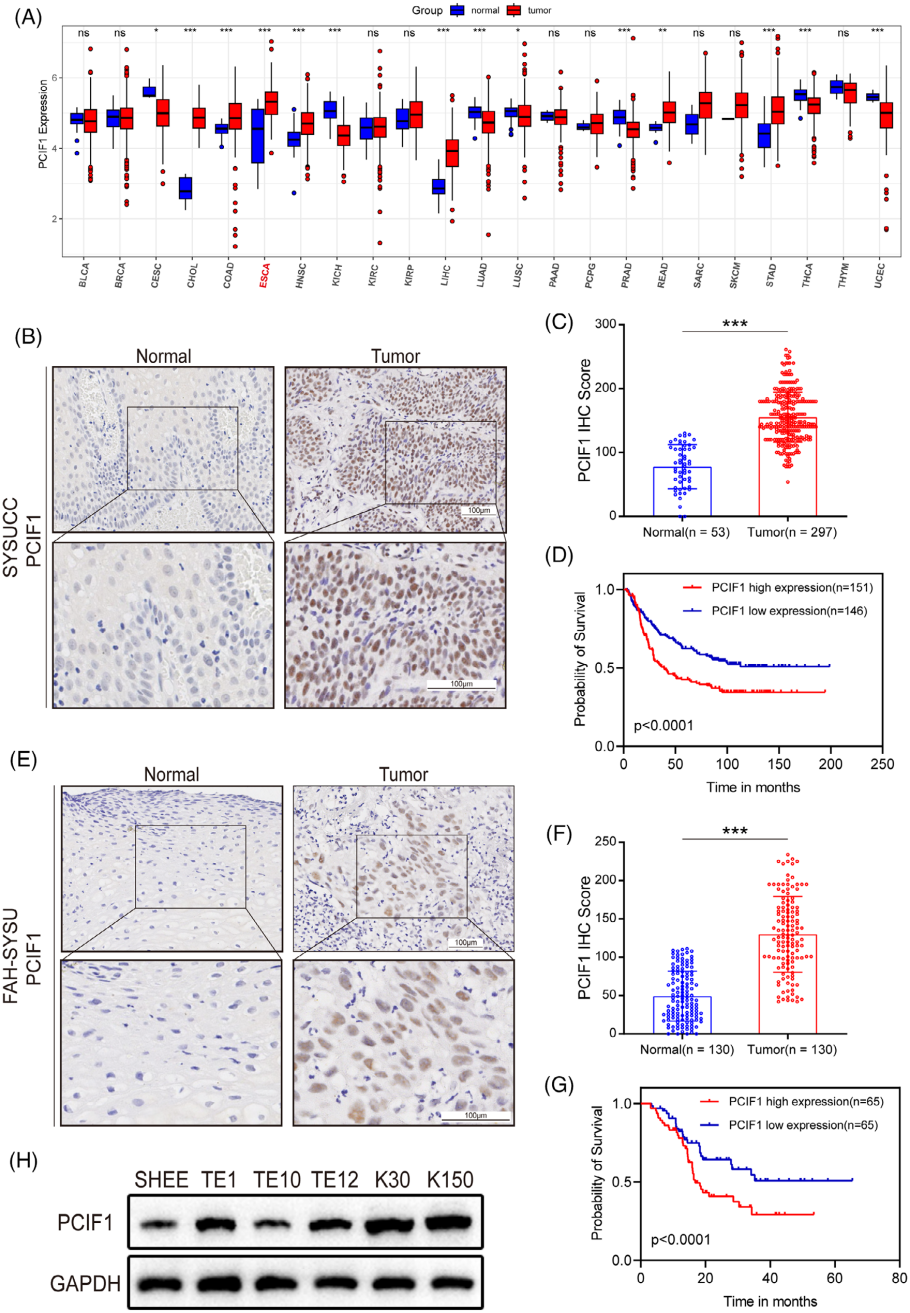
PCIF1 expression and clinical correlation in oesophageal squamous cell carcinoma (OSCC) patient samples. (A) Boxplot showing PCIF1 expression levels across TCGA cancer types, with significantly higher expression in Esophageal adenocarcinoma (ESCA) compared to normal tissues. *p* > .05, *p < .05, **p < .01, ***p < .001, Wilcoxon test. (B) Representative images of PCIF1 immunohistochemistry (IHC) staining in normal and OSCC tumour tissues from Sun Yat‐sen University Cancer Center (SYSUCC) patients' cohort. Insets show higher magnification views. Scale bar, 100 µm. (C) Quantification of PCIF1 IHC scores in normal (*n* = 53) versus tumour (*n* = 297) tissues in SYSUCC cohort, showing significantly higher PCIF1 expression in tumour samples. ***p < .001, Student's *t*‐test. (D) Kaplan–Meier survival analysis in SYSUCC cohort, demonstrating poorer survival for patients with high PCIF1 expression compared to low expression. *p* < .0001, log‐rank test. (E) Representative images of PCIF1 IHC staining in normal and OSCC tumour tissues from The First Affiliated Hospital Sun Yat‐sen University (FAH‐SYSU) patients' cohort. Insets show higher magnification views. Scale bar, 100 µm. (F) Quantification of PCIF1 IHC scores in normal (*n* = 130) versus tumour (*n* = 130) tissues in FAH‐SYSU cohort, showing significantly higher PCIF1 expression in tumour samples. ***p < .001, Student's *t*‐test. (G) Kaplan–Meier survival curves for FAH‐SYSU cohort. PCIF1 expression is higher in tumours, and high expression correlates with worse prognosis. *p* < .0001, log‐rank test. (H) Western blot analysis of PCIF1 protein levels in normal oesophageal epithelial cells (Simian Virus 40‐Immortalized Human Oesophageal Epithelial Cell Line [SHOE]) and OSCC cell lines (Oesophageal Squamous Cell Carcinoma Cell Line TE1, TE10, TE12 and Kyoto Oesophageal Squamous Cell Carcinoma Cell Line 30, 150), showing higher PCIF1 expression in OSCC cell lines.

To elucidate the clinical significance of PCIF1 in OSCC, we first analyzed its expression across various cancer types using data from the TCGA database (Figure 1A). PCIF1 was found to be significantly upregulated in several cancers, with particularly high expression observed in oesophageal cancer (Figure S1A), suggesting its potential role in tumourigenesis.

Next, we examined PCIF1 protein expression in two independent patient cohorts (SYSUCC and FAH‐SYSU) using IHC. In the SYSUCC cohort, tumour tissues exhibited markedly stronger PCIF1 staining compared to adjacent normal tissues (Figure 1B). Quantitative analysis further confirmed significantly higher IHC scores in tumour samples (Figure 1C). Consistent results were observed in the FAH‐SYSU cohort, with elevated PCIF1 staining in tumour tissues and higher IHC scores compared to normal tissues (Figure 1E,F). Besides, in the SYSUCC cohort, the elevated PCIF1 expression was associated with higher tumour grades and more advanced clinical stages (Figure S1B,C). Similarly, the FAH‐SYSU cohort demonstrated consistent findings, with higher PCIF1 levels correlating with aggressive tumour characteristics, such as advanced grade, reinforcing the role of PCIF1 in promoting OSCC progression (Figure S1E,F). In addition to its association with aggressive clinical features, PCIF1 was also linked to treatment response and recurrence involvement. Specifically, higher PCIF1 expression was observed in patients who experienced tumour recurrence and poor response to radiotherapy, as shown by increased IHC scores (Figure S1D,G).

Kaplan–Meier survival analysis consistently demonstrated that high PCIF1 expression was associated with significantly poorer overall survival in both cohorts (Figure 1D,G). Western blot analysis of PCIF1 protein levels in cell lines supported these findings. OSCC cell lines (TE1, TE9, TE12, K30 and K150) demonstrated markedly higher PCIF1 expression compared to the immortalized oesophageal epithelial cell line (SHEE) (Figure 1H). Multivariate analysis identified PCIF1 as an independent prognostic factor, further supporting its potential as a key biomarker in OSCC progression and prognosis.

### 3.2 PCIF1 regulates migration, invasion and proliferation in OSCC

**FIGURE 2:**
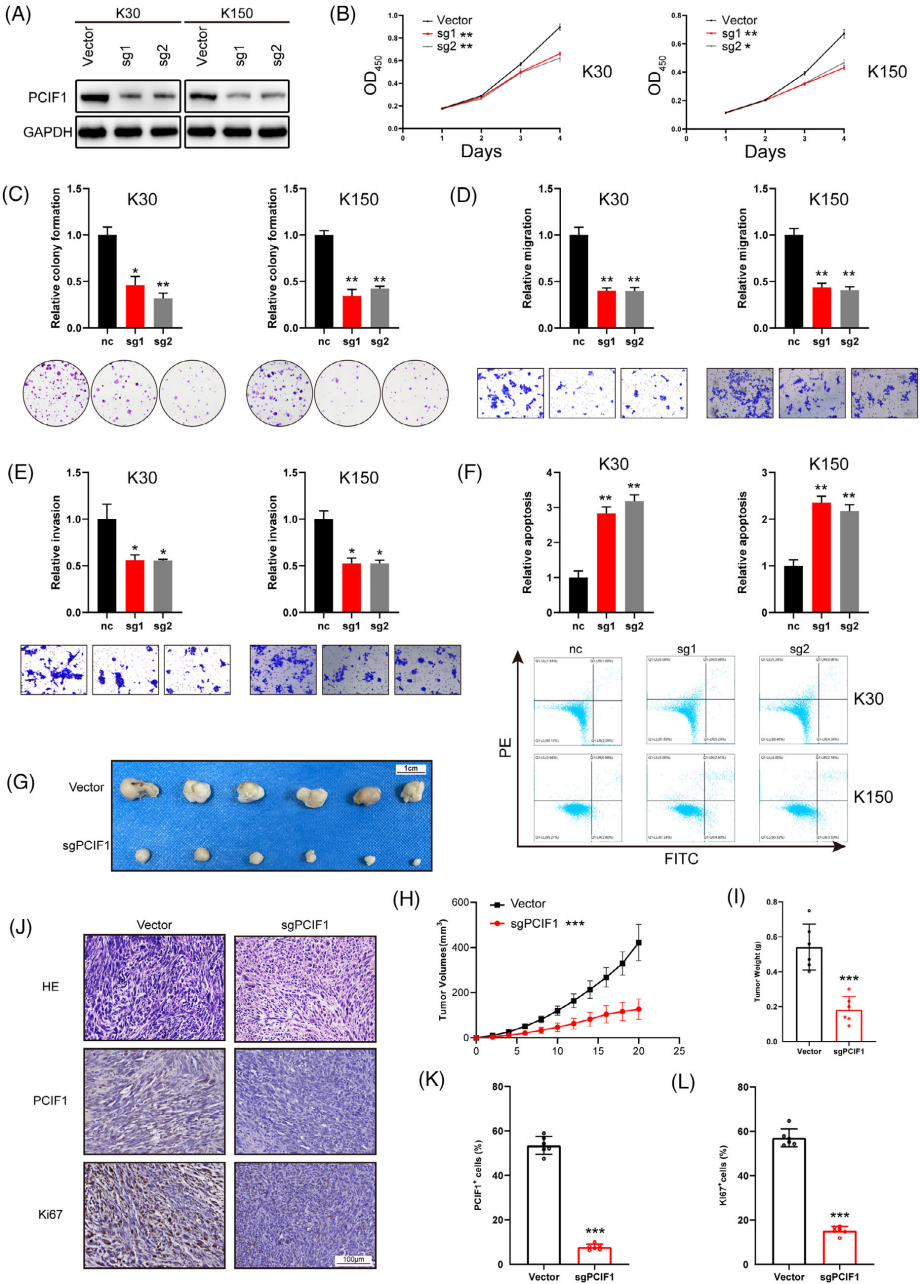
Functional role of PCIF1 in oesophageal squamous cell carcinoma (OSCC) cell proliferation, migration and invasion. (A) Western blot analysis of PCIF1 expression in different OSCC cell lines, confirming successful knockdown by sgRNAs (sg1, sg2) in KYSE30 and KYSE150 cells. (B) Cell proliferation assays showing reduced proliferation in PCIF1 knockdown cells compared to control (vector). Optical density (OD) values at 450 nm were measured at different time points. *p < .05, **p < .01 by one‐way analysis of variance (ANOVA), Dunnett's test. (C) Colony formation assays in PCIF1 knockdown cells, showing decreased colony formation relative to controls. *p < .05, **p < .01 by one‐way ANOVA with Tukey's multiple comparison test. (D) Migration assays in PCIF1 knockdown cells, showing decreased migration relative to controls. **p < .01 by one‐way ANOVA with Tukey's multiple comparison test. (E) Invasion assays demonstrating reduced invasive capabilities in PCIF1‐knockdown cells. *p < .05 by one‐way ANOVA with Tukey's multiple comparison test. (F) Increased apoptosis rates in PCIF1‐knockdown cells compared to control group. **p < .01 by one‐way ANOVA with Tukey's multiple comparison test. (G) Representative images of xenograft tumours in nude mice injected with PCIF1‐knockdown OSCC cells compared to vector controls. Scale bar, 1 cm. (H) Tumour growth curves depicting a significantly slower growth rate in mice injected with PCIF1‐knockdown cells compared to the control group. Tumour volume was measured at regular intervals. ***p < .001 by one‐way ANOVA, Dunnett's test. (I) Comparison of final tumour weights between PCIF1‐knockdown and control groups. ***p < .001, Student's *t*‐test. (J) Representative images of Haematoxylin and eosin (H&E), PCIF1 and Ki67 staining in xenograft tumours from PCIF1‐knockdown and control groups. Scale bar, 100 µm. (K) Quantification of Ki67^+^cells in PCIF1‐knockdown versus control tumours. ***p < .001, Student's *t*‐test. (L) Quantification of PCIF1^+^ cells levels in xenograft tumours, demonstrating successful knockdown with significantly lower PCIF1 levels compared to controls. ***p < .001, Student's *t*‐test.

To investigate the role of PCIF1 in OSCC, we selected KYSE30 and KYSE150 cell lines due to their notably high PCIF1 expression levels, as observed in Figure 1H. Functional assays were performed following the knockdown of PCIF1 using two specific sgRNAs (sg1 and sg2), which effectively reduced PCIF1 protein levels (Figure 2A). PCIF1 knockdown significantly reduced cell proliferation, as demonstrated by decreased optical density values in CCK‐8 assays (Figure 2B), and colony formation assays showed fewer colonies, indicating impaired proliferative capacity (Figure 2C). Migration and invasion assays revealed that PCIF1 silencing also significantly inhibited the motility of OSCC cells, suggesting a key role in promoting metastasis (Figure 2DE). Additionally, PCIF1 knockdown led to increased apoptosis, highlighting its importance in cell survival (Figure 2F). In vivo experiments further supported the tumour‐promoting role of PCIF1. PCIF1‐knockdown KYSE150 cells were injected subcutaneously into nude mice, resulting in a marked reduction in tumour volume and weight, confirming that PCIF1 promotes tumour growth (Figure 2G–I). Immunohistochemical analysis of xenograft tumours demonstrated significantly lower expression of PCIF1 and Ki67 in the PCIF1‐knockdown group, indicating reduced cell proliferation (Figure 2J–L).

To further elucidate the role of PCIF1's enzymatic activity in OSCC progression, we introduced both wild‐type PCIF1 (OE) and a catalytically inactive mutant (OE‐mut) into KYSE30 cells (Figure S2A). Overexpression of wild‐type PCIF1 significantly increased cell proliferation, as shown by higher optical density values in CCK‐8 assays (Figure S2B), along with enhanced colony formation (Figure S2C). Overexpression of PCIF1 also led to increased cell migration and invasion, whereas the catalytically inactive mutant showed diminished effects, indicating that PCIF1's enzymatic activity is critical for these processes (Figure S2DE). Apoptosis assays demonstrated reduced cell death in PCIF1‐overexpressing cells, further supporting its role in promoting OSCC progression by enhancing cell survival (Figure S2F).

Overall, these findings imply that PCIF1's enzymatic activity may play a crucial role in the aggressive features of OSCC, highlighting its potential as a target for therapeutic strategies that aim to disrupt m6Am methylation in cancer cells.

### 3.3 m6Am sequencing identifies MTF2 as a potential downstream target of PCIF1

**FIGURE 3:**
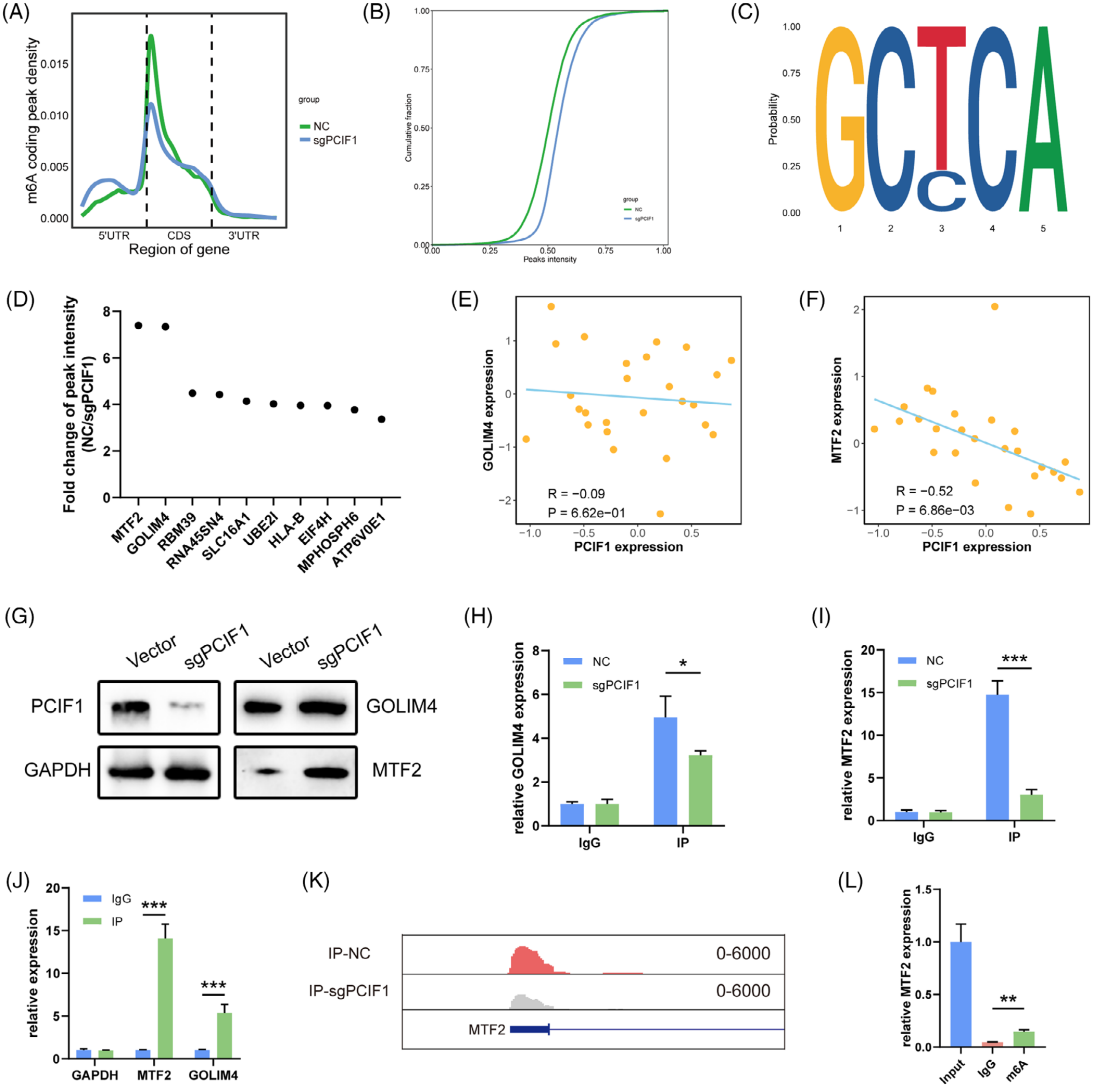
Identification of differential m6Am peaks and MTF2 as a PCIF1 target in oesophageal squamous cell carcinoma (OSCC) cells. (A) m6Am‐sequencing density plot showing peak distribution across 5′UTR, CDS and 3′UTR regions in PCIF1 knockdown and control cells. (B) Cumulative distribution of m6Am peaks in control versus PCIF1‐knockdown cells. (C) Motif enrichment analysis of top m6Am‐modified genes, identifying the GCTCA motif as significant. (D) Top 10 differentially methylated m6Am genes in PCIF1‐knockdown cells compared to controls, based on fold change. (E and F) Correlation analysis showing the correlation between PCIF1 levels and GOLIM4 levels (*R* = −.09, *p* > .05, Pearson correlation) and a strong negative correlation between PCIF1 levels and MTF2 (*R* = −.52, *p* < .01, Pearson correlation). Data were from the DepMap portal (https://DepMap.org/portal/). (G) Western blot analysis showing decreased PCIF1 protein levels and increased MTF2 expression in sgPCIF1 cells. (H and I) RNA immunoprecipitation (RIP) analysis showing PCIF1 binding to GOLIM4 and MTF2 mRNA, with a stronger interaction observed for MTF2 (*p* < .05 for GOLIM4, *p* < .001 for MTF2, Student's *t*‐test). (J) RIP‐quantitative real‐time PCR (qPCR) results confirming a significant enrichment of PCIF1‐bound MTF2 mRNA relative to GOLIM4 and GAPDH in NC cells. *p* < .001, Student's *t* test. (K) The m6Am peaks on the MTF2 gene in control and PCIF1‐knockdown OSCC cells. (L) m6A‐IP qPCR results for MTF2, indicating a reduction in m6Am modification of MTF2 mRNA upon PCIF1 knockdown. *p* < .01, Student's *t*‐test.

To further investigate the downstream effects of PCIF1‐mediated m6Am modification in OSCC, we performed m6Am sequencing in PCIF1‐knockdown and control KYSE150 cell lines. The results showed a global reduction in m6Am‐modified transcripts upon PCIF1 knockdown, with a pronounced decrease around the 5′ UTR and CDS regions, suggesting that PCIF1 specifically targets these regions for m6Am modification (Figure 3A,B). Motif analysis revealed the presence of a GCTCA motif near the m6Am sites, potentially recognized by PCIF1 (Figure 3C). Among the top 10 differentially methylated genes identified from m6Am sequencing, MTF2 and GOLIM4 emerged as potential candidates (Figure 3D). To validate these findings, we incorporated protein expression data from the DepMap portal for OSCC cell lines. A negative correlation was observed between PCIF1 expression and MTF2 protein levels (*R* = −.52, *p* < .01), whereas GOLIM4 protein levels showed no significant association (*R* = −.09, *p* = .62) (Figure 3E,F). These results suggest that PCIF1 specifically regulates MTF2 expression through m6Am modification. Western blot analysis also confirmed that PCIF1 knockdown led to a marked increase in MTF2 protein levels, whereas GOLIM4 levels remained largely unchanged (Figure 3G). RIP assays further demonstrated a direct interaction between PCIF1 and MTF2 mRNA, supporting the hypothesis that PCIF1 directly modulates MTF2 translation via m6Am modification (Figure 3H–J). Bioinformatics analysis confirmed that MTF2 mRNA contains a high‐confidence, PCIF1‐dependent m6Am site (Figure 3K).

m6A‐RIP followed by qPCR further demonstrated a significant reduction in m6Am modification on MTF2 mRNA in PCIF1‐knockdown cells, confirming the loss of m6Am methylation on MTF2 transcripts (Figure 3L). Together, these findings establish MTF2 as a key downstream target of PCIF1, mediated by m6Am modification, and highlight its potential role in OSCC progression.

### 3.4 MTF2 as a translational target of PCIF1 via m6Am modification in OSCC

**FIGURE 4:**
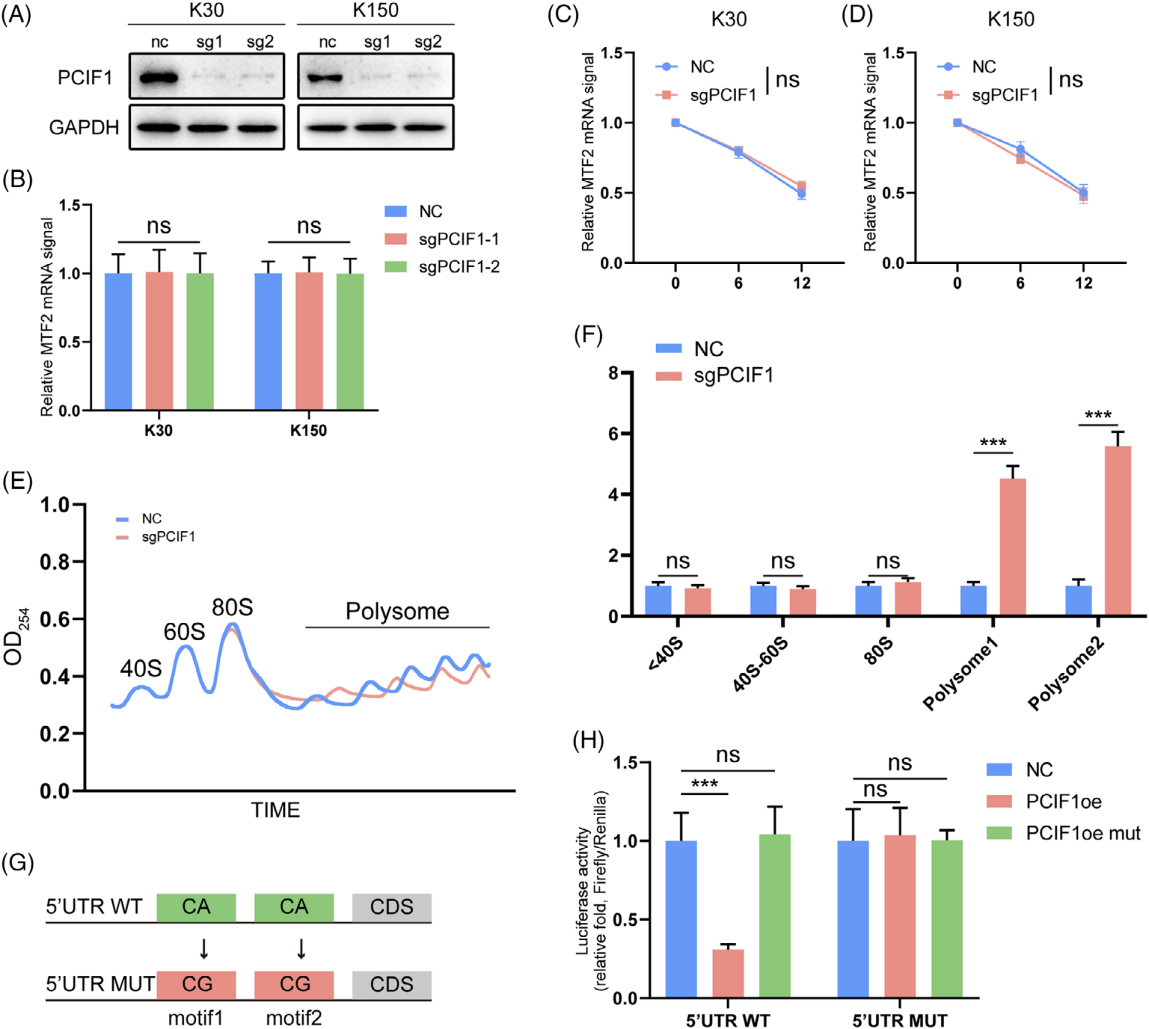
MTF2 as a direct target of PCIF1‐mediated m6Am modification in oesophageal squamous cell carcinoma (OSCC) cells. (A) Western blot analysis of MTF2 protein levels in PCIF1‐knockdown OSCC cells. (B) Quantitative real‐time PCR (qPCR) analysis of MTF2 mRNA levels in PCIF1‐knockdown OSCC cells, showing no significant change in MTF2 transcription compared to control cells. (C) mRNA stability assay of MTF2 in PCIF1‐knockdown KYSE30 cells, indicating increased mRNA stability over time, as measured by qPCR following actinomycin D treatment. (D) mRNA stability assay of MTF2 in PCIF1‐knockdown KYSE150 cells, indicating increased mRNA stability over time, as measured by qPCR following actinomycin D treatment. (E) Polysome profiling of PCIF1‐knockdown cells, showing enhanced MTF2 mRNA association with polysomes, indicating increased translation. (F) qPCR analysis of polysome fractions, confirming increased MTF2 mRNA abundance in translationally active fractions. (G) Schematic representation of mutant (mut) constructs of the MTF2 5′UTR used in the luciferase reporter assay. (H) Dual‐luciferase assay results in control (NC), PCIF1 overexpression (PCIF1 OE) and PCIF1 catalytic inactive mutant (PCIF1 OEmut) OSCC cells, transfected with either wild‐type (WT) or mut MTF2 5′UTR constructs.

Building on our previous findings, we sought to clarify the mechanism by which PCIF1 regulates MTF2 expression. Despite targeting PCIF1, there was no significant change in MTF2 mRNA levels across different cell lines (Figure 4A,B). Similarly, MTF2 mRNA stability remained unchanged, suggesting that PCIF1's regulatory effects are likely post‐transcriptional (Figure 4C,D). To further investigate this mechanism, we hypothesized that PCIF1‐mediated m6Am modification may affect MTF2 mRNA's interaction with translation initiation factors or ribosomal machinery. To further explore the translational regulation, we performed polysome profiling. The results revealed that MTF2 mRNA was significantly enriched in polysome fractions upon PCIF1 knockdown, indicating enhanced translation (Figure 4E). Subsequent qPCR analysis of MTF2 mRNA in the extracted polysome fractions further confirmed enhanced MTF2 translation following PCIF1 knockdown (Figure 4F). We further analyzed MTF2 protein expression in different PCIF1‐modified cell lines. In line with the translation assay results, PCIF1 knockout led to a marked increase in MTF2 protein levels, whereas overexpression of wild‐type PCIF1 decreased MTF2 expression (Figure S3A–C). Importantly, overexpression of catalytically inactive PCIF1 failed to affect MTF2 protein levels, underscoring the necessity of PCIF1's methyltransferase activity in regulating MTF2 translation (Figure S3A–C).

We next employed a 5′ UTR luciferase reporter assay to dissect the mechanism of PCIF1‐mediated translational control. In PCIF1‐knockout cells, luciferase activity was significantly higher when the wild‐type MTF2 5′ UTR construct was transfected, while the mutant construct showed no response, confirming that PCIF1 specifically regulates MTF2 translation via its 5′UTR (Figure 4G,H). These results collectively confirm that PCIF1 negatively regulates MTF2 translation via m6Am modification in OSCC, implicating the PCIF1‐m6Am‐MTF2 axis as a potential pathway driving tumour progression.

### 3.5 Functional role and clinical implications of MTF2 in OSCC

**FIGURE 5:**
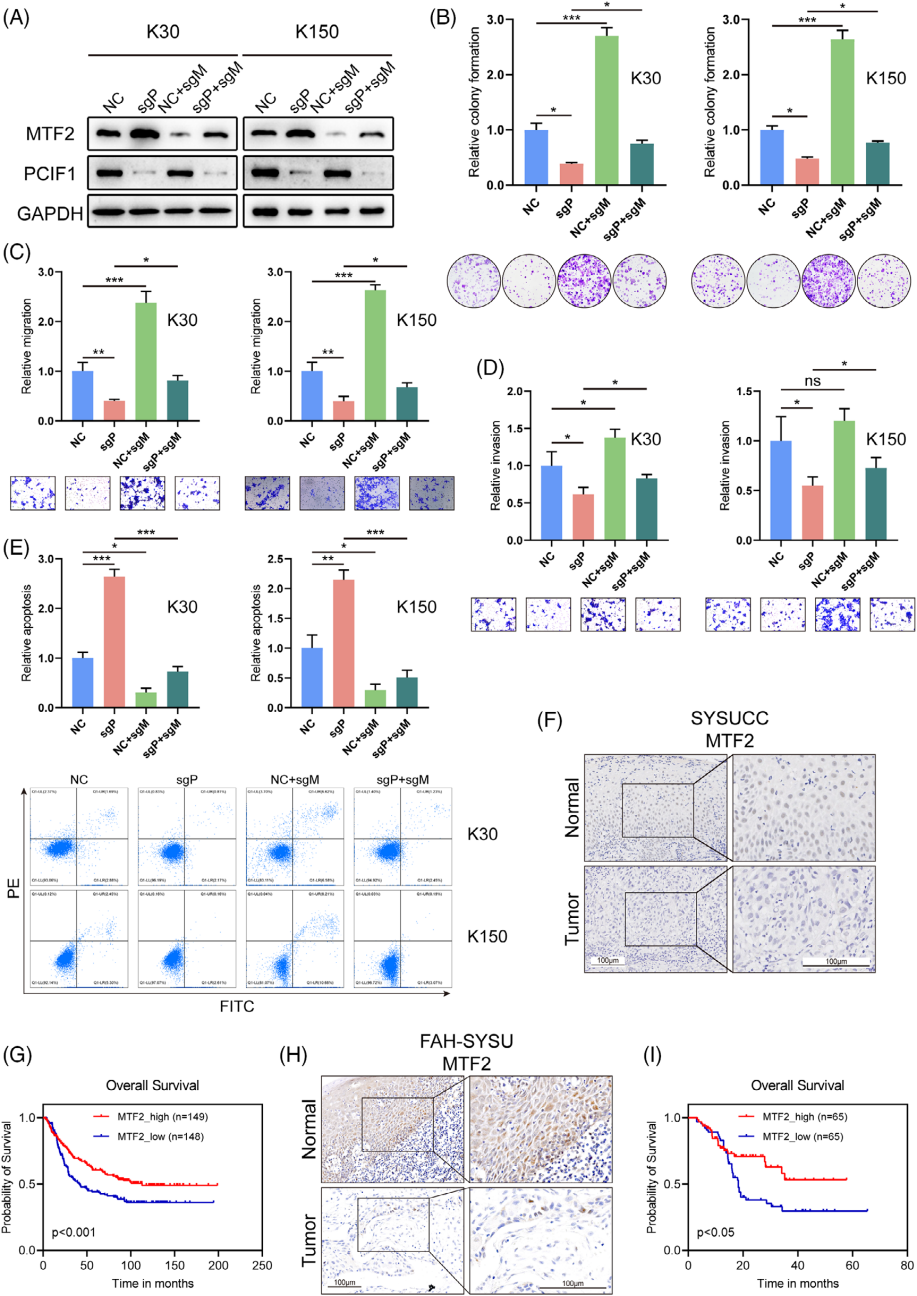
Rescue experiments confirming PCIF1‐MTF2 axis in oesophageal squamous cell carcinoma (OSCC) cells. (A) Western blot analysis of MTF2 and PCIF1 protein levels in KYSE30 (K30) and KYSE150 (K150) cells. (B–E) Functional assays (colony formation, migration, invasion and apoptosis) showing that co‐knockdown of MTF2 restores proliferation (B), migration (C) and invasion (D), while reducing apoptosis (E) in PCIF1‐knockdown cells. *p* > .05, *p < .05, ***p < .01 by one‐way analysis of variance (ANOVA) with Tukey's multiple comparison test. (F and G) Representative immunohistochemistry (IHC) staining images (F) and Kaplan–Meier survival analysis (G) for MTF2 expression in the SYSUCC ESCC patient cohort. (H and I) Representative IHC staining images (H) and Kaplan–Meier survival analysis (I) for MTF2 expression in the FAH‐SYSU OSCC patient cohort. *p* < .001, *p* < .05, log‐rank test.

To further validate the role of MTF2 as a downstream target of PCIF1 and its clinical relevance in OSCC, rescue experiments were performed in KYSE30 and KYSE150. Co‐knockdown of PCIF1 and MTF2 reversed the suppressive effects of PCIF1 knockdown on cell proliferation, migration and invasion, restoring these tumorigenic capabilities to levels similar to the control group (Figure 5B–E). This indicates that PCIF1 may exert its oncogenic functions partly through MTF2 downregulation.

Subsequent immunohistochemical analysis of OSCC patient samples revealed a consistent pattern of low MTF2 expression correlating with worse clinical outcomes, mirroring the trends observed for PCIF1 (Figure 5F). In both cohorts, reduced MTF2 levels were associated with higher tumour grade, advanced stage, recurrence and poor treatment response (Figure S4A–F). Moreover, Kaplan–Meier survival analysis showed that patients with low MTF2 expression had significantly lower overall survival compared to those with high MTF2 expression, confirming its prognostic value (Figure 5H,J). These results collectively underscore MTF2's role as a tumour suppressor in OSCC and suggest that PCIF1‐mediated suppression of MTF2 contributes to tumour progression and adverse clinical features.

### 3.6 PCIF1‐MTF2 axis drives OSCC tumourigenesis in transgenic mouse models

**FIGURE 6:**
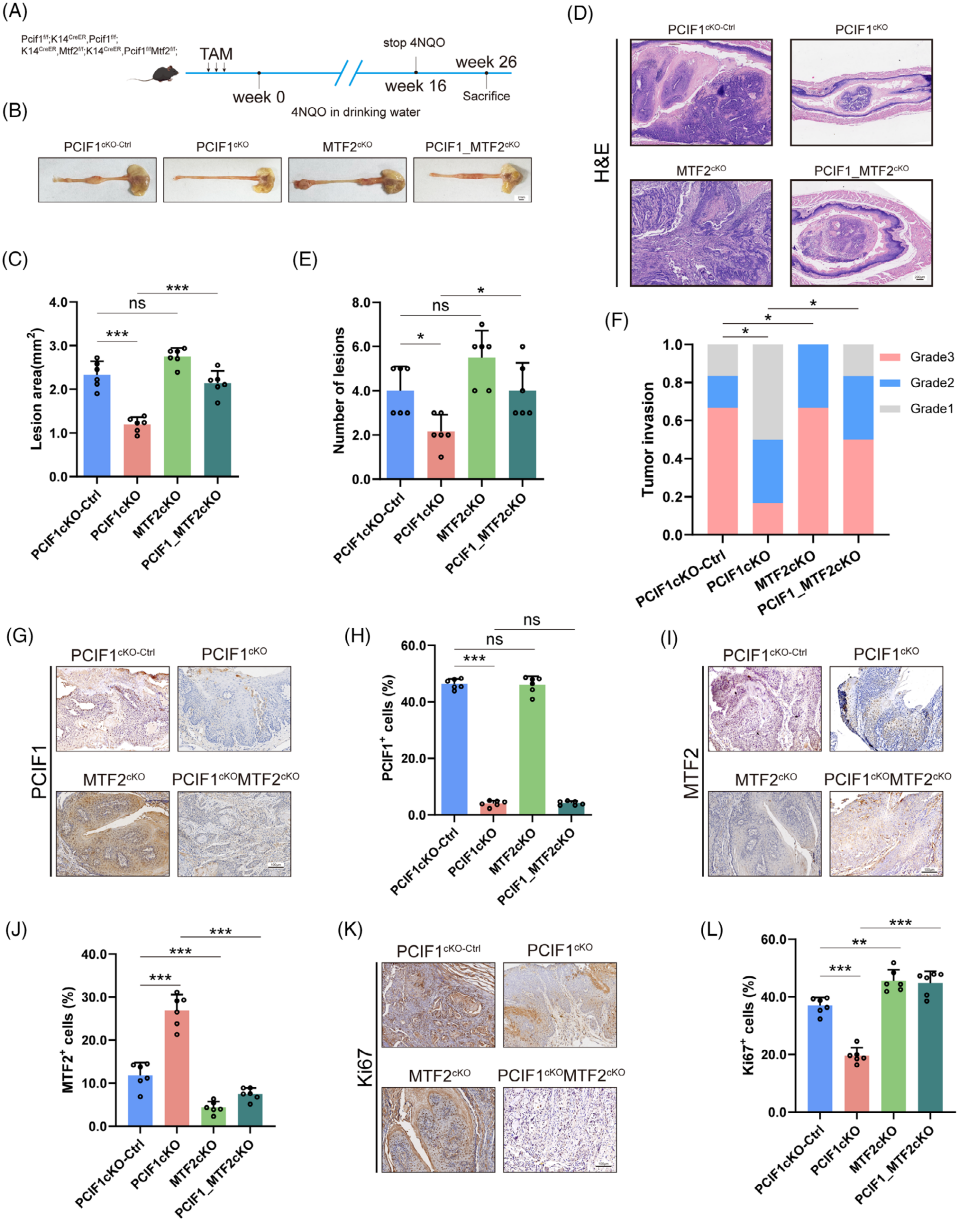
Transgenic mouse models validating PCIF1‐MTF2 axis in oesophageal squamous cell carcinoma (OSCC) tumourigenesis. (A) Schematic representation of the experimental design for generating transgenic mouse models with conditional knockouts of PCIF1 and MTF2 in oesophageal epithelial cells (K14‐CreERT2). (B) Representative images of oesophageal tumours from different knockout groups: PCIF1 knockout alone, MTF2 knockout alone and PCIF1/MTF2 double knockout. Scale bar, 2 mm. (C) Quantification of tumour lesions area across the mouse groups, showing that PCIF1 knockout significantly reduces tumour incidence, whereas concurrent MTF2 knockout restores tumour formation. Data are presented as mean ± standard deviation. *p* > .05, ***p < .01 by one‐way analysis of variance (ANOVA) with Tukey's multiple comparison test. (D) Haematoxylin and eosin (H&E) staining of oesophageal tissues from different transgenic mouse groups, demonstrating increased tumour aggressiveness and invasiveness in the PCIF1/MTF2 double‐knockout mice compared to PCIF1‐knockout mice. Scale bar, 200 µm. (E) Quantification of tumour burden, measured by the number of lesions, indicating that the loss of MTF2 reverses the tumour‐suppressive effect of PCIF1 knockout. *p* > .05, *p < .05 by one‐way ANOVA with Tukey's multiple comparison test. (F) Histological analysis of tumour grades, illustrating the enhanced tumour severity in PCIF1/MTF2 double‐knockout mice. *p < .05 by Pearson chi‐square test. (G and H) Representative immunohistochemistry (IHC) images (G) and quantification (H) of PCIF1 expression in oesophageal tissues from different transgenic mouse groups. Scale bar, 100 µm. *p* > .05, ***p < .001 by one‐way ANOVA with Tukey's multiple comparison test. (I and J) Representative IHC images (I) and quantification (J) of MTF2 expression in oesophageal tissues from different transgenic mouse groups. Scale bar, 100 µm. ***p < .001 by one‐way ANOVA with Tukey's multiple comparison test. (K and L) Representative IHC images (K) and quantification (L) of KI67 expression in oesophageal tissues from different transgenic mouse groups. Scale bar, 100 µm. **p < .01, ***p < .001 by one‐way ANOVA with Tukey's multiple comparison test.

To investigate the in vivo role of the PCIF1‐MTF2 axis in OSCC tumourigenesis, we generated transgenic mice models with conditional knockouts of PCIF1, MTF2 or both genes in oesophageal epithelial cells. These transgenic models were created by breeding PCIF1‐floxed and MTF2‐floxed mice with K14‐CreERT2 mice, ensuring specific gene deletions in oesophageal tissues. Following tamoxifen induction, these mice were treated with 4‐nitroquinoline 1‐oxide (4NQO) for 16 weeks to induce OSCC (Figure 6A). Histological analysis at Week 26 revealed that PCIF1 knockout significantly reduced tumour lesion size and incidence, suggesting its critical role in tumour initiation and growth suppression (Figure 6B,C). In contrast, MTF2 knockout led to increased tumour size, whereas the combined knockout of both PCIF1 and MTF2 further exacerbated tumour formation, demonstrating a compensatory oncogenic effect in the absence of MTF2 (Figure 6D–F). H&E staining confirmed varying levels of tumour invasion across different genotypes, with double‐knockout mice exhibiting more aggressive tumour phenotypes compared to PCIF1 knockout alone (Figure 6D–F). IHC analyses showed that PCIF1 deletion was associated with reduced expression of Ki67, a proliferation marker, whereas MTF2 deletion increased Ki67 expression, highlighting MTF2's tumour‐suppressive role (Figure 6K,L). The expression of PCIF1 and MTF2 in tumour tissues was consistent with these findings: PCIF1 loss decreased PCIF1^+^ cell populations, whereas MTF2 loss increased MTF2^+^ cells (Figure 6G–J). These results reinforce the pivotal role of the PCIF1‐MTF2 axis in modulating OSCC progression and underscore MTF2's role as a tumour suppressor, potentially counteracting PCIF1's oncogenic effects.

### 3.7 PCIF1 knockout potentiates anti‐PD1 immunotherapy in OSCC

**FIGURE 7:**
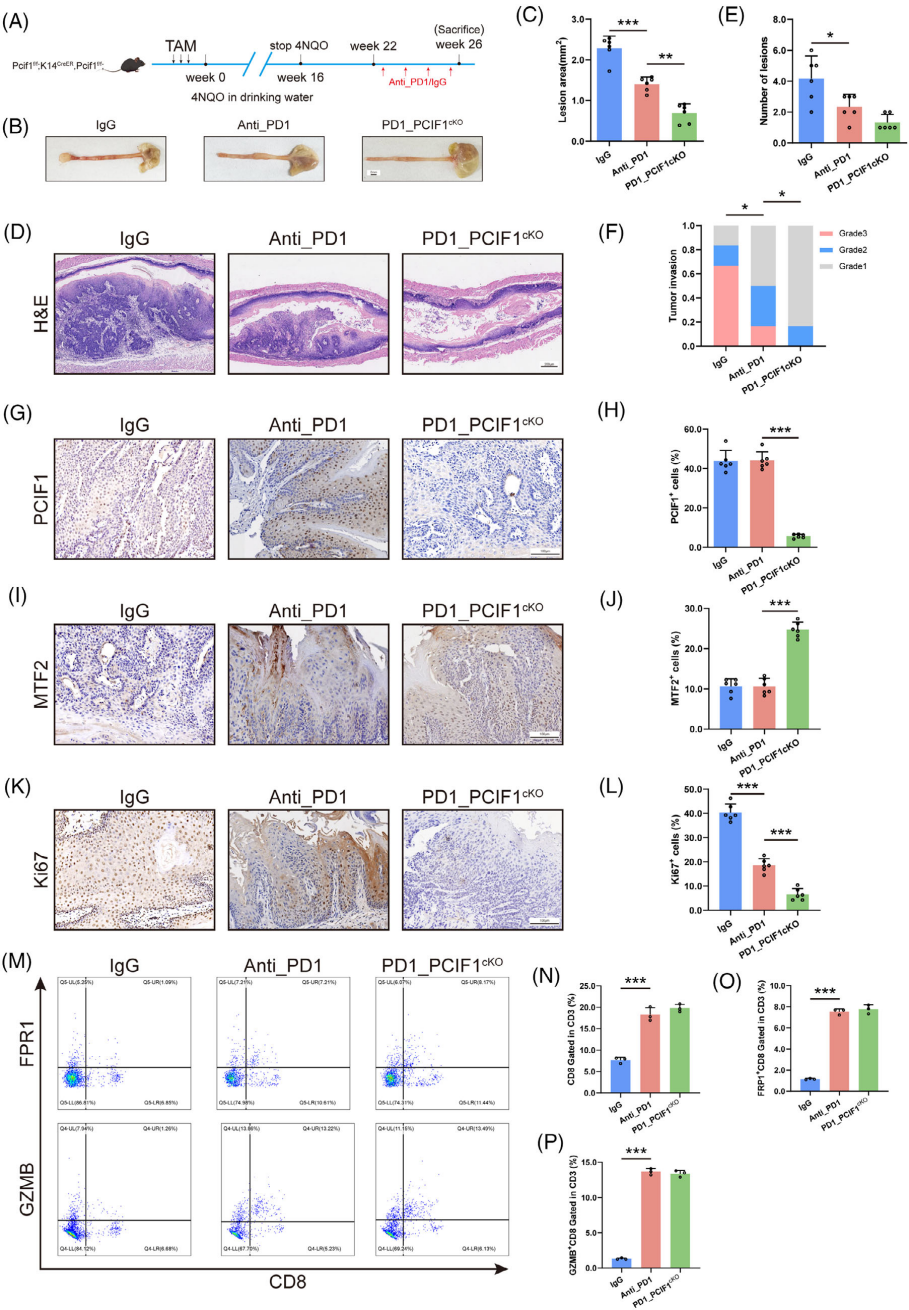
PCIF1 knockout enhances the efficacy of anti‐PD1 therapy in oesophageal squamous cell carcinoma (OSCC). (A) Schematic of the experimental design involving the generation of PCIF1 knockout in OSCC mouse models, followed by treatment with anti‐PD1 antibody or IgG control. Mice were administered 4NQO in drinking water to induce tumour formation. Anti‐PD1 or IgG treatment was administered starting from Week 22, and mice were sacrificed at Week 26 for analysis. (B) Representative images of oesophageal tumours from different treatment groups: IgG control, Anti‐PD1 treated and combined anti‐PD1 + PCIF1 knockout. Scale bar, 2 mm. (C) Quantification of lesion area, showing a reduction in tumour size in the PCIF1 knockout group and in the combined anti‐PD1 + PCIF1 knockout group compared to controls. **p < .01, ***p < .001 by one‐way analysis of variance (ANOVA) with Tukey's multiple comparison test. (D) Haematoxylin and eosin (H&E) staining of oesophageal tissue sections from each treatment group. Scale bar, 200 µm. (E) Quantification of the number of lesions, indicating a significant reduction in the combined treatment group. *p < .05 by one‐way ANOVA with Tukey's multiple comparison test. (F) Tumour invasion grade analysis, showing decreased aggressiveness in the anti‐PD1 + PCIF1 knockout group. *p < .05 by Pearson chi‐square test. (G and H) Immunohistochemistry (IHC) staining (G) and quantification (H) of PCIF1‐positive cells across the treatment groups, showing a marked reduction in PCIF1 expression in the knockout group. Scale bar, 100 µm. ***p < .001 by one‐way ANOVA with Tukey's multiple comparison test. (I and J) IHC staining (I) and quantification (J) of MTF2‐positive cells, with increased MTF2 expression in the Anti‐PD1 + PCIF1 knockout group. Scale bar, 100 µm. ***p < .001 by one‐way ANOVA with Tukey's multiple comparison test. (K and L) IHC staining (K) for Ki67 with quantification (L) showing decreased Ki67‐positive cells in the anti‐PD1 and PCIF1 knockout groups, particularly in the combined treatment group, indicating reduced tumour proliferation. Scale bar, 100 µm. ***p < .001 by one‐way ANOVA with Tukey's multiple comparison test. (M–P) Flow cytometry analysis showing no significant changes in CD8^+^ T cell function across the treatment groups, including FPR1+ and GZMB+ CD8^+^ T cells, suggesting that PCIF1 knockout and anti‐PD1 treatment operate via distinct mechanisms. *p* > .05, ***p < .001 by Student's *t*‐test.

To investigate whether targeting PCIF1 could enhance the efficacy of anti‐PD1 immunotherapy in OSCC, we conducted a comparative analysis of tumour progression in PCIF1‐knockout mice treated with either anti‐PD1 antibody or IgG control (Figure 7A). In comparison to anti‐PD1 monotherapy, the combination of PCIF1 knockout with anti‐PD1 treatment significantly reduced tumour burden (Figure 7B), as evidenced by decreased lesion area and a reduced number of tumour lesions (Figure 7C and E). Histopathological evaluation revealed that tumours in the combination group were of lower histological grade and exhibited less invasive characteristics, suggesting that PCIF1 deficiency enhances the tumour‐suppressive effects of PD1 blockade (Figure 7D,F). Mechanistically, immunohistochemical analysis demonstrated that PCIF1 knockout combined with anti‐PD1 treatment not only reduced the proliferation marker Ki67 but also led to a pronounced upregulation of the tumour suppressor MTF2 (Figure 7G–L). Specifically, while anti‐PD1 therapy alone modestly decreased Ki67 levels, the combination with PCIF1 knockout further suppressed cellular proliferation, implying that the absence of PCIF1 sensitizes tumours to immunotherapy. Moreover, the increase in MTF2 expression observed in the PCIF1 knockout group suggests a potential mechanism by which PCIF1 inhibition augments the therapeutic impact of PD1 blockade, possibly by restoring MTF2‐mediated suppression of oncogenic pathways (Figure 7I,J).

Interestingly, flow cytometric analysis of CD8^+^ T cells showed no significant differences in effector molecule expression, such as granzyme B (GZMB) and perforin (PRF1), between the PCIF1 knockout group and the IgG control group (Figure 7M–P). This suggests that PCIF1's tumour‐suppressive effects are independent of direct immune modulation. Instead, PCIF1 knockout appears to enhance anti‐PD1 efficacy by reducing tumour aggressiveness rather than altering the immune microenvironment, suggesting a novel mechanism for sensitizing tumours to immunotherapy. Together, these findings demonstrate that PCIF1 depletion and PD1 inhibition act through complementary but mechanistically distinct pathways to suppress tumour growth. This highlights the potential of dual targeting PCIF1 and PD1 as a novel therapeutic strategy for OSCC, leveraging their synergistic effects to maximize anti‐tumour efficacy.

## 4 DISCUSSION

In this study, we identified the PCIF1‐MTF2 axis as a significant pathway driving OSCC progression through m6Am modifications, building on the broader landscape of RNA methylation in cancer biology. Our findings demonstrate that PCIF1 selectively represses MTF2 translation, leading to tumour progression, which aligns with the role of m6Am modifications as previously reported in several cancers, such as colorectal cancer, glioblastoma and gastric cancer. For instance, recent studies have shown that PCIF1‐mediated m6Am modification selectively inhibits mRNA translation of tumour suppressors, leading to enhanced cell proliferation and metastasis.^12^ Similarly, in colorectal cancer, PCIF1 has been found to stabilize transcript stability in response to anti‐immunotherapy.^16^ These studies underscore the importance of PCIF1 in stabilizing oncogenic mRNAs across various cancers, suggesting its broader role as an oncogenic factor.

Interestingly, the impact of PCIF1 on chromatin regulators, such as MTF2, emphasizes a novel mechanism where RNA methylation influences epigenetic landscapes.^26^, ^27^ MTF2 is essential for maintaining histone H3K27me3 modifications that suppress oncogene expression.^23^ Previous research has also indicated that MTF2 downregulation is linked to increased cell proliferation and metastasis in cancers like leukaemia and colorectal cancer, where its loss leads to chromatin instability and the activation of oncogenic pathways. Our data suggest that PCIF1‐mediated m6Am modifications directly inhibit MTF2 translation, creating a feedback loop that disrupts chromatin‐mediated gene repression, thereby promoting tumour progression.

The selective effect of PCIF1 on MTF2 translation, without altering global protein synthesis, is a crucial finding. This suggests a fine‐tuning mechanism where PCIF1 selectively targets specific tumour suppressors through m6Am methylation. Ribosome profiling analysis confirmed that PCIF1 knockout reduces ribosome occupancy on MTF2 mRNA, further supporting its role in regulating MTF2 translation. In contrast, no significant changes in global ribosome distribution or overall translational efficiency were observed, reinforcing the specificity of PCIF1's effect. Furthermore, RIP assays demonstrated a direct interaction between PCIF1 and MTF2 mRNA, providing strong evidence for PCIF1's role in selectively modulating MTF2 translation. This specificity has been observed in other studies, where PCIF1 selectively modulated m6Am on key transcripts without affecting the broader transcriptome.^12^, ^28^ Such specificity indicates that PCIF1's role in oncogenesis may be context dependent, involving a precise interaction with the cellular environment and existing gene regulatory networks.

The clinical relevance of PCIF1 and MTF2 as biomarkers is further reinforced by our study. The observed correlation between PCIF1 overexpression and poor prognosis aligns with previous findings, suggesting that PCIF1 can serve as a prognostic marker across multiple malignancies. Furthermore, MTF2 downregulation has been associated with advanced stages and poor outcomes in various cancers,^24^, ^29^ indicating its role as a common tumour suppressor. Given that MTF2 restoration in our rescue experiments reversed the oncogenic effects of PCIF1 knockdown, targeting the PCIF1‐MTF2 axis could offer a promising therapeutic strategy, not only in OSCC but also in other cancers where this pathway is dysregulated.

The in vivo evidence provided by the transgenic mouse models validates the functional impact of the PCIF1‐MTF2 axis on tumour development. Similar transgenic models have been used to demonstrate the role of RNA methylation in cancers, where m6A regulators were shown to promote tumour progression through mRNA stabilization.^30^, ^31^, ^32^ Our results align with these studies, highlighting the importance of RNA modifications in vivo and their potential as therapeutic targets. Specifically, PCIF1 knockout in our mouse models significantly reduced tumour incidence, supporting the notion that inhibiting PCIF1 could offer therapeutic benefits by restoring MTF2 function. Previous study demonstrated the impact of PCIF1 on mRNA methylation and its implications for immune checkpoint therapy, providing foundational insights into its regulatory role in cancer.^16^ Notably, our data further confirm that PCIF1 knockout significantly enhances the efficacy of PD‐1 immunotherapy in OSCC, suggesting a synergistic effect that highlights the therapeutic potential of PCIF1 inhibition alongside immune checkpoint blockade. Interestingly, flow cytometry analysis revealed that PCIF1 knockout does not affect the function of CD8^+^ T cells, as evidenced by unchanged levels of effector molecules. This indicates that the anti‐tumour effect of PCIF1 depletion operates through mechanisms independent of direct modulation of the immune microenvironment. Emerging studies suggest that tumour burden reduction, particularly through modulating oncogenic pathways, can indirectly enhance the efficacy of immune checkpoint inhibitors.^33^ ^34^ The TME in aggressive malignancies is often immunosuppressive, characterized by high tumour burden, metabolic competition and recruitment of immune‐suppressive cells such as regulatory T cells and myeloid‐derived suppressor cells.^35^ By reducing tumour burden, PCIF1 depletion may help shift the TME towards a state that is more permissive to immune attack, thereby enhancing PD‐1 blockade efficacy. Similar findings have been reported in other cancers, where oncogene inhibition led to increased T‐cell infiltration and improved responses to immunotherapy.^36^, ^37^

While our study provides novel insights into the PCIF1‐MTF2 axis in OSCC, several limitations remain. First, the precise mechanism by which m6Am modification suppresses MTF2 translation requires further investigation, particularly its impact on translation initiation. Second, additional PCIF1‐regulated targets may exist beyond MTF2, warranting transcriptome‐wide analyses such as CLIP‐seq or Ribo‐seq. Third, although our in vivo models support the therapeutic potential of PCIF1 inhibition, its clinical applicability requires further validation through the development of selective PCIF1 inhibitors and their evaluation in preclinical models. Lastly, a more comprehensive analysis of the tumour immune microenvironment using scRNA‐seq or multiplex IHC could provide deeper insights into PCIF1's broader role in tumour progression.

In conclusion, this study provides a comprehensive analysis of the PCIF1‐MTF2 axis in OSCC, elucidating its role in tumour progression through m6Am modifications. By linking RNA methylation to chromatin regulation, our findings open new avenues for exploring RNA modifications as therapeutic targets. Future research should focus on developing specific inhibitors of PCIF1, potentially in combination with epigenetic therapies aimed at restoring MTF2 function, to effectively manage OSCC and possibly other cancers influenced by similar regulatory mechanisms.

## AUTHOR CONTRIBUTIONS

Kang Li, Yuxuan Yi, Rongsong Ling, Caihua Zhang, Zhihui Zhang, Ganping Wang, Maosheng Cheng, Shuang Chen conceived and designed the experiments. Kang Li, Yuxuan Yi, Yue Wang and Rongsong Ling conducted most of the experiments, including cell line studies, m6Am sequencing and transgenic mouse experiments, while Caihua Zhang, Zhihui Zhang and Maosheng Cheng handled data collection, statistical analysis and interpretation of results. Jie Chen provided essential technical support for biochemical assays and helped revise the manuscript. Shuang Chen provided oversight, funding and supervision of the project, contributing to the conceptual development. Kang Li, Yuxuan Yi, Rongsong Ling, Caihua Zhang, Ganping Wang and Maosheng Cheng drafted the manuscript. Kang Li, Yuxuan Yi and Rongsong Ling are co‐first authors, and Shuang Chen is co‐corresponding author. All authors have read and approved the final manuscript, and contributed significantly to this study.

## CONFLICT OF INTEREST STATEMENT

The authors declare no conflicts of interest.

## ETHICS STATEMENT

All patients had informed consent and had not received preoperative radiotherapy or chemotherapy. All animal experiments in this study were approved by the Institutional Animal Care and Use Committee, Sun Yat‐sen University (IACUC, SYSU).

## Supporting information



Supporting information

## Data Availability

The m6Am sequencing data supporting this study are available in the Genome Sequence Archive at the National Genomics Data Center under accession number HRA010202. Additional datasets and resources can be accessed via the DepMap portal (https://DepMap.org/portal/) and cBioPortal for Cancer Genomics (https://www.cbioportal.org/) under the TCGA PanCancer Atlas Studies.
