# Supplementary material for: PCIF1 drives oesophageal squamous cell carcinoma progression via m6Am‐mediated suppression of MTF2 translation
Source: Clin Transl Med. 2025 Mar 28;15(4):e70286. doi: 10.1002/ctm2.70286 (PMC11953057; doi:10.1002/ctm2.70286)
Supplement: Supplementary file 1 — Supporting information [file CTM2-15-e70286-s001.docx]

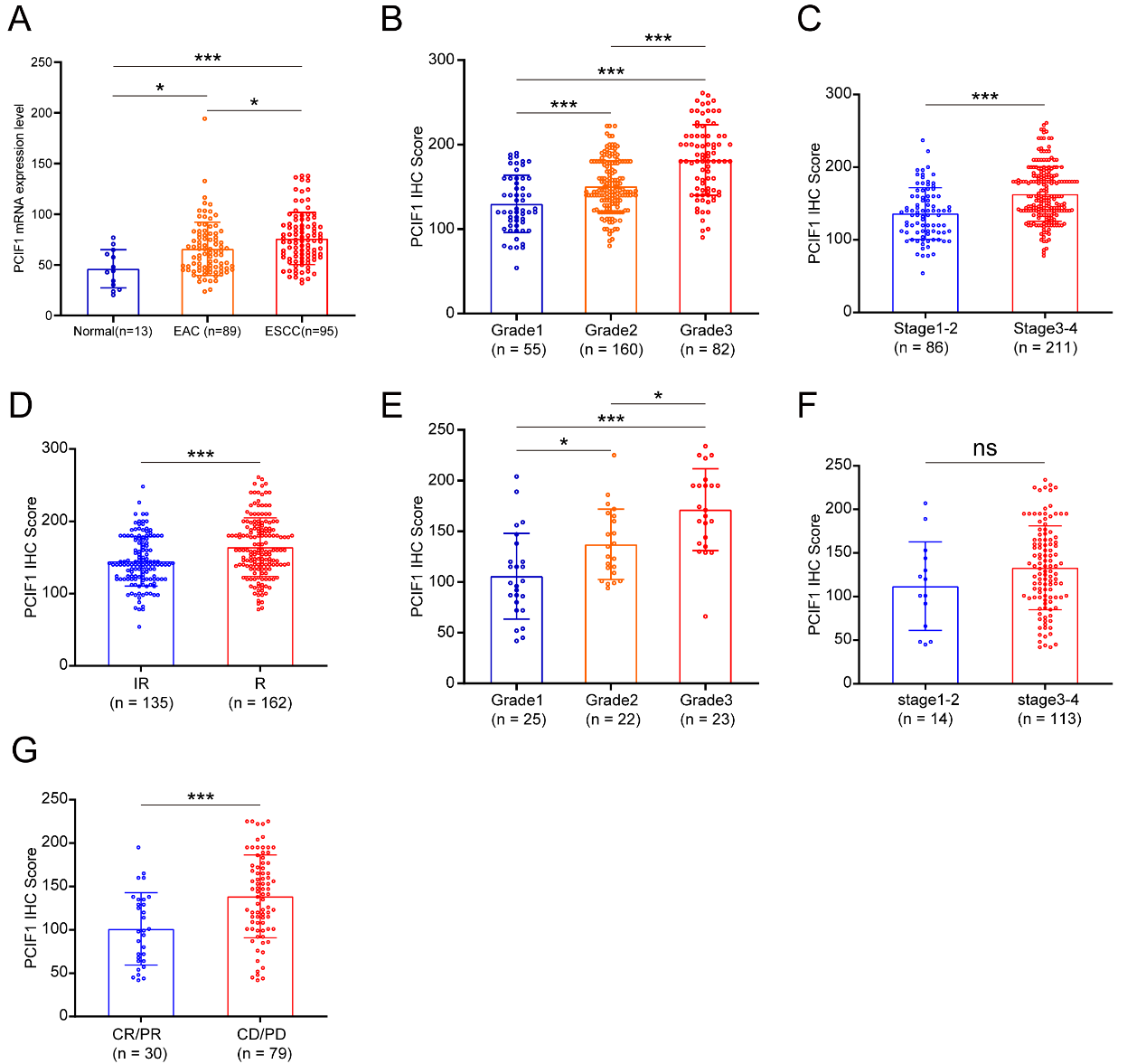


Supplemental Figure 1 Related to Figure 1. PCIF1 expression correlates with clinical features in ESCC patients.

(A) PCIF1 mRNA expression levels in normal esophageal tissues versus primary ESCC tumors.

(B) PCIF1 IHC scores in tumors stratified by histological grade (Grade 1, Grade 2, and Grade 3) from SYSUCC cohort.

(C) PCIF1 IHC scores in tumors classified by clinical stage (Stage 1-2 vs. Stage 3-4) from SYSUCC cohort.

(D) PCIF1 IHC scores in patients with no recurrence (IR) versus recurrence (R) from SYSUCC cohort.

(E) PCIF1 IHC scores in tumors stratified by pathological grade from FAH-SYSU cohort.

(F) PCIF1 IHC scores in patients classified by early (Stage 1-2) versus advanced stages (Stage 3-4) in FAH-SYSU cohort.

(G) PCIF1 IHC scores in patients with complete/partial response (CR/PR) versus those with clinical disease progression (CD/PD) after therapy in FAH-SYSU cohort.


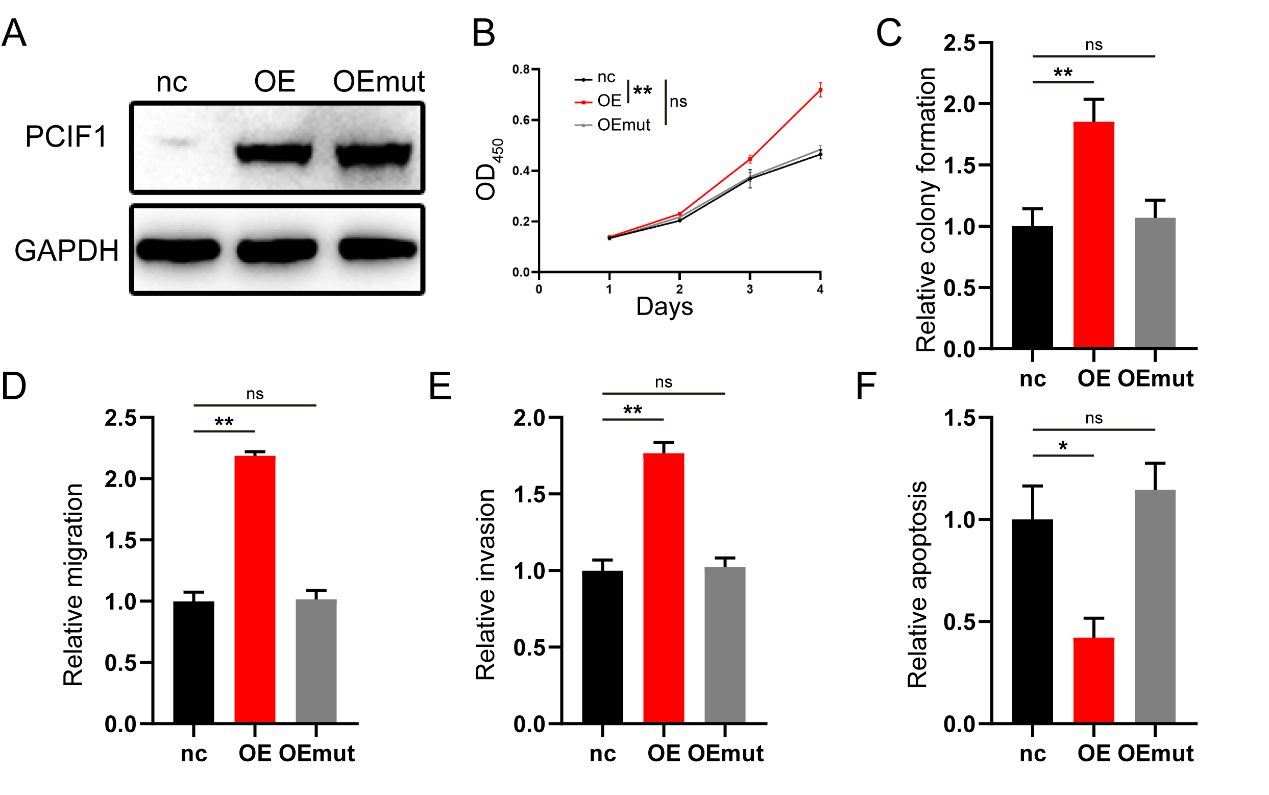


Supplemental Figure 2 Related to Figure 2. PCIF1 overexpression promotes ESCC cell progression.

(A) Western blot analysis of PCIF1 expression in ESCC cells transfected with control, wild-type PCIF1 (OE), and mutant PCIF1 (OE-mut), confirming successful overexpression.

(B) Cell proliferation assay in ESCC cells, indicating increased proliferation only in wild-type PCIF1 overexpression compared to mutant PCIF1.

(C) Colony formation assay showing enhanced colony numbers in wild-type PCIF1-overexpressing cells, while mutant PCIF1 showed no significant effect.

(D) Migration assay results revealing increased migratory capacity in wild-type PCIF1-overexpressing cells, but not in mutant PCIF1-overexpressing cells.

(E) Invasion assay results demonstrating that wild-type PCIF1 promotes cell invasion, while mutant PCIF1 does not.

(F) Apoptosis assay results showing reduced apoptosis only in wild-type PCIF1-overexpressing cells, with no effect in mutant PCIF1-overexpressing cells.


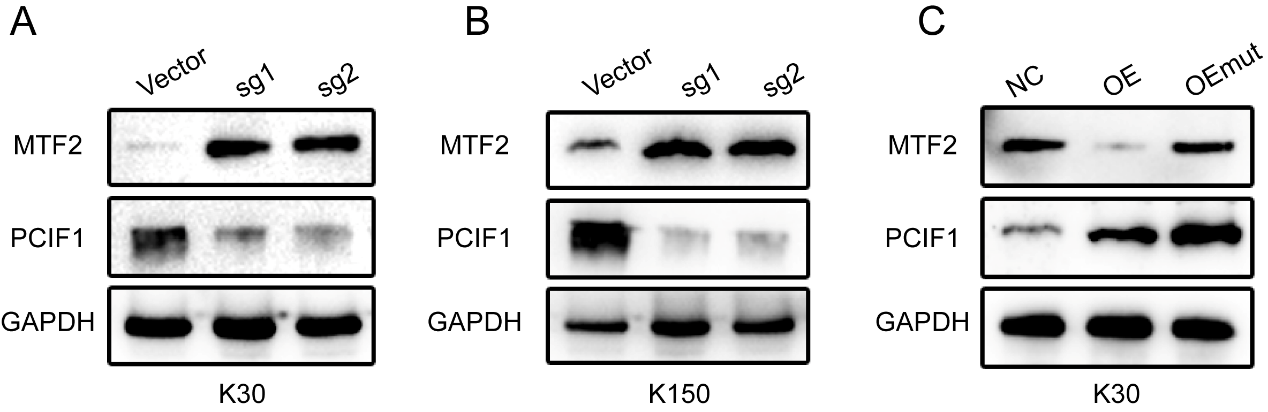


Supplemental Figure 3 Related to Figure 4. PCIF1 regulation of MTF2 protein levels.

1. Western blot analysis of MTF2 expression in PCIF1-knockdown ESCC cells, indicating increased MTF2 protein levels after PCIF1 depletion.
2. Western blot analysis of MTF2 expression in PCIF1-knockdown ESCC cells, indicating increased MTF2 protein levels after PCIF1 depletion.

(C) Western blot analysis of MTF2 expression in wild-type PCIF1-overexpressing and mutant PCIF1 overexpression ESCC cells, showing reduced MTF2 protein levels compared to control.


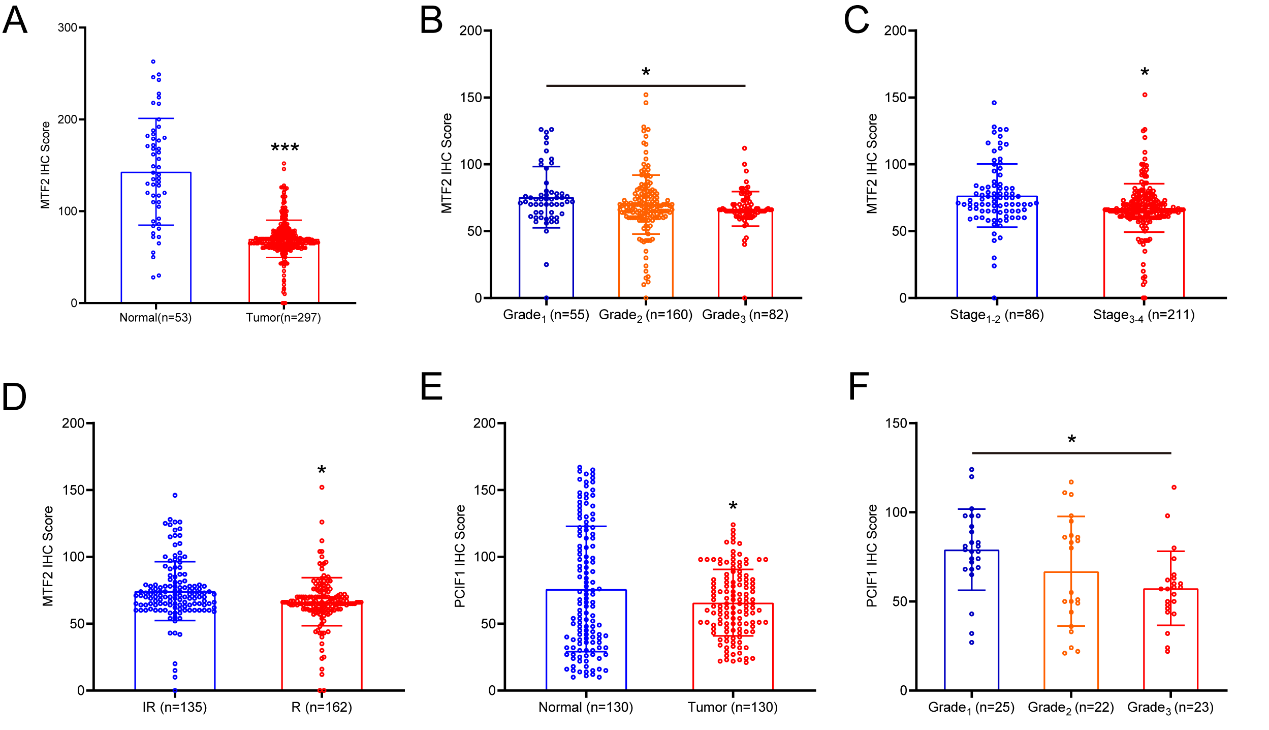


Supplemental Figure 4 Related to Figure 5. Correlation of MTF2 expression with clinical characteristics in ESCC.

(A-D) Immunohistochemistry (IHC) analysis of MTF2 expression in SYSUCC cohort, comparing normal esophageal tissues to ESCC tissues (A), and stratifying by tumor grade (B), stage (C), and recurrence (D) showing reduced MTF2 expression in advanced stages and higher grades.

(E-F) IHC analysis of MTF2 in FAH-SYSU cohort, comparing normal esophageal tissues to ESCC tissues (E). Lower MTF2 levels are observed in patients with higher tumor grades (F).

Supplemental Table 1: The demographic, pathologic, and clinical information of SYSUCC cohort.

| Patient chracteristics | No. (%) |
| --- | --- |
| Age (yr) | 58.51 |
| < 50 | 59 (19.9%) |
| ≥ 50 | 238 (80.1%) |
| Gender |  |
| Male | 233 (78.5%) |
| Female | 64 (21.5%) |
| T stage |  |
| T1 | 15 (5.1%) |
| T2 | 71 (23.9%) |
| T3 | 200 (67.3%) |
| T4 | 11 (3.7%) |
| Tumor grade |  |
| G1(Well differentiated) | 55 (18.5%) |
| G2(Moderately differentiated) | 160 (53.9%) |
| G3(Poorly differentiated) | 82 (27.6%) |
| Treatment response and recurrence |  |
| YES | 135(45.5%) |
| NO | 162(54.5%) |
| Survival Status |  |
| Alive | 140 (47.1%) |
| Dead | 157 (52.9%) |

Supplemental Table 2: The demographic, pathologic, and clinical information of FAH-SYSU cohort.

| Patient chracteristics | No. (%) |
| --- | --- |
| Age (yr) | 60.71 |
| < 50 | 11 (8.5%) |
| ≥ 50 | 119 (91.5%) |
| Gender |  |
| Male | 112 (86.2%) |
| Female | 18 (13.8%) |
| T stage |  |
| T1 | 4 (3.0%) |
| T2 | 10 (7.7%) |
| T3 | 63 (48.5%) |
| T4 | 50 (38.5%) |
| Not avaiable | 3 (2.3%) |
| Tumor grade |  |
| G1(Well differentiated) | 25 (19.2%) |
| G2(Moderately differentiated) | 22 (16.9%) |
| G3(Poorly differentiated) | 23 (17.7%) |
| Not avaiable | 60 (46.2%) |
| Efficacy evaluation of radiotherapy |  |
| CR/PR | 30(23.1%) |
| CD/PD | 79(60.8%) |
| Not avaiable | 21 (16.1%) |
| Survival Status |  |
| Alive | 69 (53.1%) |
| Dead | 61 (46.9%) |

Supplemental Table 3: Primer and oligonucleotides sequences.

| Oligonucleotides | sequence |
| --- | --- |
| h-MTF2-Forward Primer | 5’- TCAAACGTCTACCATTACAGTGG-3’ |
| h-MTF2-Reverse Primer | 5’-TCCAGGGTGCAATCTATCCCA-3’ |
| h-GAPDH-Forward Primer | 5’-AGATCCCTCCAAAATCAAGTGG-3’ |
| h-GAPDH-Reverse Primer | 5’- GGCAGAGATGATGACCCTTTT-3’ |
| h-PCIF1-Forward Primer | 5’- AATCGTCCCTACTACTTCAACCG-3’ |
| h-PCIF1-Reverse Primer | 5’- GGTCCGAAATCACATCGTGC-3’ |
| h-GOLIM4-Forward Primer | 5’- CCCTCTCCGCCCAGTTACA-3’ |
| h-GOLIM4-Reverse Primer | 5’- CTCCTCGTGTTGGCTTTTCA-3’ |
| sg-MTF2 | 5’-ATCACACTCGAGTCAATATG-3’ |
| sg-PCIF1-1 | 5’- CACCTAGCGGTAAAGGAGCCACTG-3’ |
| sg-PCIF1-2 | 5’- CACCCGGTTGAAAGACTCCCGTGG-3’ |
